# Implementing Nonlethal Solutions for Free-Roaming Cat Management in a County in the Southeastern United States

**DOI:** 10.3389/fvets.2019.00259

**Published:** 2019-08-22

**Authors:** Francis Hamilton

**Affiliations:** Behavioral Sciences Collegium, Eckerd College, St. Petersburg, FL, United States

**Keywords:** free-roaming cat management, TNVR, animal control and management, nonlethal methods of animal control, social change for animals

## Abstract

From 2006 to 2017, stray or free-roaming cats ranged from 35 to 54% of all animals going into the public shelter in Hillsborough County, Florida. Shelter overcrowding of cats, including free-roaming, feral, or community cats, is a major problem in parts of the world. Issues with free-roaming cats include the welfare of the cats themselves, public health and zoonotic diseases, spread of diseases to other species or pet cats, public nuisance, and predation of wildlife. Animal control is a government function and ultimately a taxpayer issue. This paper describes three methods of humane, nonlethal management of free-roaming cat populations that were successfully applied in Hillsborough County, Florida: low-income spay/neuter vouchers; small- and large-scale trap, neuter, vaccinate, and return (TNVR); and return to field (RTF). The methods used were contrary to the long-accepted practice of using euthanasia to control cat populations and generated opposition among certain stakeholders. While the human population of the county increased by 14.6% from 2010 to 2017, the methods used to control free-roaming cats assisted in achieving a 51% decrease in intake since 2007 and increased the live-release rate to 81.8% of cats taken in at the Pet Resources Center in 2017. This paper examines how this change in intake was achieved despite opposition to these programs.

## Introduction

Governmental agencies are responsible for controlling the excess population at public animal shelters (1). A major part of the excess consists of unlicensed, free-roaming cats, sometimes also referred to as *feral* (unsocialized) *cats, community cats* (which may be owned but unlicensed), and *strays*. This paper will use the term *free-roaming cats*. Free-roaming cats are any cats, whether owned, stray, or feral, that are free to roam the streets. There are a variety of estimates of the number of free-roaming cats in the United States. The highest estimate is 60–100 million; a more conservative estimate is 30–45 million (2). These cats can produce litters of 1–6 kittens and on average have kittens 1.6 times a year (3).

Attempts to manage the number of free-roaming cats have to balance multiple objectives: protect the welfare of the cats themselves, control threats to public health and constrain zoonotic disease, prevent the spread of disease to other species or to pet cats, and avoid nuisance and the predation of wildlife (2). In addition, the local governments responsible for implementing management programs have to find the money to pay for them (1, 4, 5).

This paper reports empirical results from a study of three nonlethal free-roaming cat management programs undertaken by the only open-access animal shelter in Hillsborough County and two non-profits in southwest Florida, where citizens and community organizations were able to significantly decrease shelter intake and increase the live-release rate. This location was selected for several reasons. First, animal control functions in Florida are a county responsibility. Second, the shelter chosen was the only open-admissions shelter in the county during the study period and because it was a government shelter, data were readily available about costs and the numbers of animals in the shelter. Third, this local community had the highest companion animal euthanasia rate in Florida (6, 7). Finally, the programs described were specifically targeted to the geographical area of the study.

When it became evident at the beginning of 2000 that the euthanasia rate for cats in the Hillsborough County Animal Services (HCAS) shelter was over 90%, private citizens and the Humane Society of Tampa Bay (HSTB) took steps to introduce a new approach. Although trap, neuter, vaccinate, and return (TNVR) had been practiced on a small scale in the county, local, state, and federal officials, including the Florida Fish and Wildlife Commission, claimed that TNVR was against state law. Opponents cited Florida Statutes 828.12 (cruelty to animals), 828.13 (abandonment), 379.231 (releasing non-native species in the wild), and 372.265 (regulation of foreign animals) and Florida Administrative Code 68A-4.005, aimed at wildlife and birds. These statutes were used to intimidate citizens and municipal agencies with the implication that they made TNR illegal. In fact, except for 828.12 and 13, these laws only applied to wildlife, not domestic animals. Florida Statutes 828.12 and 13 have since been interpreted by county governments not to be aimed at TNVR.

There was nonetheless a history of local initiatives. A local TNVR organization helped neuter cats and had a small sanctuary. In addition to opening a low-cost clinic that operated Monday to Friday, HSTB conducted a small clinic once a month to sterilize free-roaming cats. After it was founded in 2001, the Animal Coalition of Tampa (ACT) established a monthly all-volunteer clinic to sterilize up to 100 free-roaming cats at a time in borrowed veterinary clinics. In 2002, a county voucher program to assist individuals with the cost of spaying and neutering began to target people in poverty. In 2006, ACT opened a free-standing clinic (high quality, high volume, spay/neuter, HQHVSN) modeled after the Humane Alliance clinic in North Carolina (8). ACT then offered daily no-reservation free-roaming cat surgeries while continuing its once-a-month all-volunteer clinic for free-roaming cats. Both clinics served two underserved market segments: demographical and behavioral. Low income families have been identified by Chu et al. (9) as having a lower percentage of cats being neutered (51.4% as opposed to 90.7–96.2% for higher incomes). Benka and McCobb (10) and White et al. (11) found that a large number of owned cats had never seen a veterinarian with the main reason given as “too expensive.” These two clinics met those needs for affordability and accessibility.

In 2002, a conference was held in Tampa to address the high rate of euthanasia of cats in Hillsborough County, with a follow-up conference in 2003. Finally, in April 2004, No More Homeless Pets in Hillsborough County (NMHP-HC) was established, bringing together HCAS, HSTB, ACT, Big Cat Rescue, and more than 35 other smaller rescue and animal rights groups to “end euthanasia as a primary means of animal population control and enhance the quality of life for dogs and cats in Hillsborough County” (12). The organization held quarterly meetings and started to benchmark the data collected by constituent groups about the treatment of cats in the county.

Separately, at the end of 2006, the American Society for the Prevention of Cruelty to Animals (ASPCA) announced a new national program called Mission Orange. It promised “intensive efforts on humane care and protection” in four cities, one of which was Tampa [(13), p. 3], where $600,000 was pledged over a three-year period to complement shelter adoption programs and to fund a larger number of targeted spay/neuter surgeries for dogs and for both owned and free-roaming cats.

## Background

Hillsborough County (1,052 square miles) is located at the midway point on Florida's west coast. There are three incorporated municipalities including Tampa, but most of the county is unincorporated. The population of the county is 1,408,566 (14) and has been growing steadily, by 19.8% from 1990 to 2000 and by 17.6% from 2000 to 2007. After slowing during the recession, it recovered and grew by a further 14.6% from 2010 to 2017.

A majority of the population lives in the urban part of the county, with only 3.5% living in census-defined rural areas. The population is 17% black and 28% Hispanic. The county is fourth in the state and fifty-ninth nationally for the value of its farm products (15). Approximately 15% of the population is at or below the poverty level. There are 580,323 housing units in the county (14).

These data point to substantial socioeconomic, cultural, and linguistic diversity in the local population, factors to which effective programs for cat management need to be sensitive (16). Nationally, the largest group of unaltered and free-roaming pets is to be found in areas of poverty, which also have the most limited availability of veterinary services (17, 18). The continuous flow of both people and their companion animals into the county meant that unless some way could be found to reduce the number of free-roaming cats entering the Hillsborough County animal shelter, the euthanasia rate of over 90% would persist. In 2005, for example, 19,936 free-roaming cats entered the shelter and only 1,345 (4.6%) survived. In 2007 there was a slight improvement as 18,637 entered the shelter and 1,837 (6.3%) survived.

## Data Sources and Methods

The data for the analysis that follows come from a variety of sources. Some is based on participant observation. The author was a member of the county Animal Advisory Committee for 8 years and a cofounder of both No More Homeless Pets–Hillsborough County (NMHP-HC) and the Animal Coalition of Tampa (ACT). Data from HCAS, later renamed Pet Resources (PRC) in 2014, are also used, including budgetary and workload information. Other documentary sources include the minutes of meetings held by all the agencies involved. Field notes consisting of interviews, audio and video recordings, text and tape from all three agencies and e-mails have also been used.

## Three Targeted Programs

Three targeted programs have been used over time to try to lower the intake of free-roaming cats at the Hillsborough County shelter to a point where the management focus could shift from the routine warehousing and euthanasia of animals to increasing live-release rates (LRR).

### Low-Income Vouchers

Low-income voucher programs are Hillsborough County's oldest formal cat population control mechanism. They were pioneered in New Hampshire in 1994 and then spread to other states, cities, and counties (1). Most such programs across the country use federally established low-income program guidelines to qualify applicants, who must be enrolled in one of seven income-based programs (section 8 Housing; Medicaid; Temporary Assistance for Needy Families; Supplemental Security Income; Women, Infants, and Children; or the Supplemental Nutrition Assistance Program). Eligibility is established using verifiable documentation by agencies separate from the county animal control service. The programs have generally been successful in bringing down rates of animal intake and euthanasia at shelters (1, 19).

In 1981 Hillsborough County established a subsidized spay-neuter program whereby citizens who had their animals sterilized at a veterinarian's office could apply for a $20 rebate. The subsidy did not target low-income people and the majority of the people who took advantage of it were middle-income (B. Armstrong, personal communication, 2002).

The shift toward the New Hampshire model targeting the poor and away from the rebate was initiated in 2001 by the county Animal Advisory Committee (AAC):

This committee advises and makes recommendations to the Board of County Commissioners [BOCC] and the Hillsborough County Pet Resources Department on issues concerning long-range plans [and] general policies [for] shelter programs and services in the County. Additionally, it advises the BOCC and county administrations regarding the revisions to the Animal Ordinance, animal-related resolutions, and policies concerning companion animals in Hillsborough County ^1^.

The Spay/Neuter Voucher Program (SNVP) established by the Hillsborough BOCC in 2002 provided sterilization surgery, a rabies vaccination, and a county license tag for a $10 copay. The SNVP replaced the earlier subsidized program. It was funded by the differential license fees charged to owners of intact animals. The fee reimbursements for male and female dogs and cats were set by the Hillsborough County Veterinary Medical Society (HCVMS) and have not been raised since 2002. A financial analysis of HCAS annual reports from 1997 to 2011 shows that the average cost per surgery to the county under this program is ~$65 per animal. In contrast, in 1997 it cost the county $168 to catch, house, and dispose of an animal^2^.

In the first year of the program, a number of issues arose about application procedures and how to cover additional costs for blood tests and other requirements demanded by some veterinarians. Those added requirements increased the $10 copay by hundreds of dollars in some cases. In 2004 and 2005 about 2,000 vouchers were used each year. There were also disagreements between HCVMS and the county over whether non-profit clinics (HSTB and ACT) had the right to perform voucher surgeries. Following a decision that the two non-profit clinics could participate in the program along with any for-profit veterinary clinic in the county, eventually non-profit clinics performed a majority of the surgeries. The Hillsborough Animal Health Foundation (HAHF), the educational arm of the HCVMS, established a third non-profit clinic in 2013. In 2018, 11 out of 125 clinics in the county were participating in the program, with the three non-profit clinics accounting for 67% of the surgeries performed (S. Trebatoski, personal communication, July 26, 2018).

The two non-profit clinics also played a key role in early promoting and marketing of the program. For example, they followed up with people who had applied for vouchers but did not use them, finding that HCAS rejected some applications because some low-income individuals could not properly fill out the form. They also realized that some low-income people worked when the clinics were open and could not afford to take time off from work to bring their pets in for surgery. Therefore, the clinics adjusted their surgery days and hours. One clinic also developed a transportation unit to help low-income people get to the clinic because buses did not allow pets on board. The number of vouchers redeemed increased from 3,000 in 2008 to almost 6,000 in 2009.

The demand for the program was so high that HCAS projected it would not have enough money from license tag sales to fund the program and stopped issuing vouchers between May and October 2010. After the program resumed with money from reserve funds, the County Administrator sent a letter to the Animal Advisory Committee asking for a recommendation on who could conduct a study to determine the number of targeted sterilizations that would be needed annually to sustain the reductions in impounds experienced since the SNVP was implemented (20). The results of this study along with the tasks of developing a feasible financial plan and minimizing the “administrative and geographic hurdles” encountered by users were incorporated into the charge for the HC Animal Services Task Force (21).

**Figure 1:**
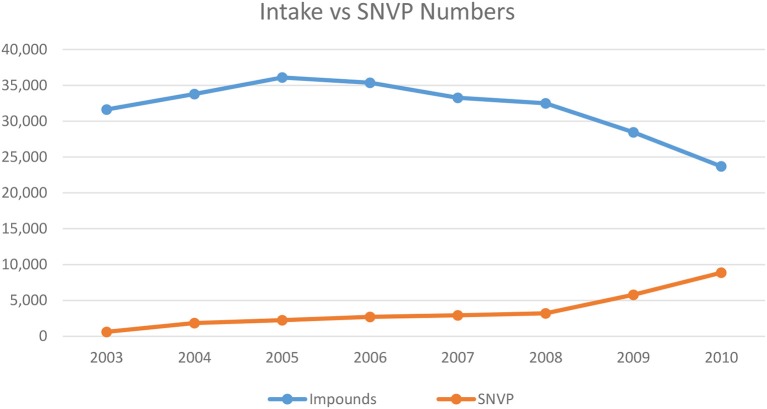
Chart developed by Marsh (17) to display SNVP surgeries to HCAS intake.

Peter Marsh, who had helped to establish the New Hampshire program, was retained to make this assessment. He recommended that the program should try to subsidize 7,500 surgeries a year, and since that time the actual number has varied between 6,000 and 7,500 (22). Marsh argued that there were a number of factors that affected the impoundment of free-roaming cats at the shelter, such as the discontinuation of proactive trapping of stray cats by HCAS, a reduction in HCAS shelter hours, and the initiation of a policy to charge a surrender fee for owned cats. Nevertheless, Marsh wrote, “It appears that the SNVP has played a significant role in reducing HCAS impounds” and there is an inverse correlation (*r* = −0.85) between the decrease in intakes to the shelter and the number of redeemed vouchers. Figure 1 displays the chart he provided to the committee (23).

Although the Spay/Neuter Voucher Program has had some success in bringing down the intake numbers and subsequent number of euthanized cats, it is not in and of itself sufficient to achieve the desired results (18, 24–27). For example, it is aimed only at cats owned by citizens whose income is at or below the poverty level. It is true that many of those cats are free roaming, but there is really no way to tell whether some of the cats treated through the program might actually be feral cats, strays, or unowned free-roaming cats. The majority of cats entering HCAS are labeled as “strays” (35–54% of all animals entering the shelter; Table 1). Other programs were developed to address those cats.

**Table 1:** Cat intake as a percentage of total impounds, 2005–2017.

**Calendar year**	**Total intake, all animals**	**Owned cats**	**Stray cats**	**Total cat intake**
CY 2017	18,293	8.95%	41.07%	9,151
CY 2016	16,434	9.57%	38.43%	7,889
CY 2015	14,792	9.25%	35.42%	6,607
CY 2014	16,376	6.01%	44.20%	8,223
CY 2013	20,614	6.53%	48.75%	11,063
CY 2012	20,198	5.56%	46.88%	10,591
CY 2011	20,405	5.21%	47.87%	10,831
CY 2010	21,913	5.75%	50.78%	12,388
CY 2009	26,966	7%	54%	15,041
CY 2008	30,895	15%	45%	18,432
CY 2007	31,699	17%	42%	18,637
CY 2006	34,191	15%	40%	19,139
CY 2005	34,485			19,936

*Source: Hillsborough County Animal Services/PRC monthly reports*.

### Trap-Neuter-Vaccinate-Return (TNVR): Beyond Small-Scale Efforts

The first trap-neuter-return organization in Hillsborough County, Fix, and Feed Feral, was incorporated in 1997. It was a small, all-volunteer organization in the northern part of the county that trapped and sterilized a small number of free-roaming cats and then returned them to the places where they were caught. It also had a barn sanctuary for cats that could not be returned.

Individuals and groups who wanted to practice TNVR in Hillsborough County in the early 2000s faced several challenges. They needed, first, to find veterinary clinics willing and able to handle free-roaming cats, a process that requires extra safety steps. Of the approximately 116 veterinary clinics in Hillsborough County at the time, fewer than 10 would admit free-roaming cats, and even fewer offered a discount to fix a free-roaming cat. A second challenge was cost, because even under the best of circumstances neutering can cost over $100 per cat. The third challenge was timing. Even though a clinic might be willing take a free-roaming cat, appointments are required at clinics and most free-roaming cats cannot easily be caught and fitted to normal clinic schedules. There is also a challenge involving the traps used to capture the cats: although Home Depot and Lowe's, for example, carry raccoon traps, which can be used for cats, they are not cheap and most people would not purchase such a trap unless they planned to catch more than one cat.

Some history is in order here. When HSTB opened a low-cost spay/neuter clinic in 2000 it performed 21 surgeries a day. During its monthly spay/neuter clinic for free-roaming cats it would accept up to 35 animals (J. Wagner, personal communication, 2001). The surgeries were organized on a private clinic model with a single veterinarian. Cats would be dropped off early in the morning and picked up later in the afternoon. These low-cost surgeries enabled some management of the population of animals owned by low-income families.

Then, in 2001 a new organization, the Animal Coalition of Tampa (ACT), was founded in Hillsborough County. It held once-a-month Spay Days beginning in January 2002 at various private clinics around the county. Modeled after the Feral Cat Coalition in San Diego (28), it was an all-volunteer effort, with multiple veterinarians, technicians, and lay assistants giving their time one Sunday a month. In their first full year (2002) they sterilized and ear-tipped 706 free-roaming cats. They also provided traps and training for caretakers. The traps were originally located in nine different depots around the county in volunteers' homes. Caretakers would make an appointment and then be sent to the closest depot to pick up their traps. If they did not know how to use them, volunteers would give them brief instructions on how to trap the cats. After Spay Day, the caretakers would return the traps to the depot.

But this was a small-scale operation. Extrapolating from Levy et al. (25), 12% of the households in a given geographical area feed a mean of 3.6 cats each. Based on US census data for households in Hillsborough County, this means that there were more than 210,000 free-roaming cats in the county. Thus, even the combined efforts of HSTB, ACT, and Fix and Feed Feral would be insufficient to slow the flow of cats and kittens into the HCAS shelter. The county needed to move from small-scale efforts to larger ones.

A step toward operating on a larger scale was taken when ACT opened a HQHVSN clinic to provide services for both owned and free-roaming cats. The Humane Alliance clinic in North Carolina began exporting its expertise in 2005. The ACT clinic in Hillsborough County was the ninth clinic to emulate the North Carolina original and the first to open in a populated urban area. After opening in March 2006, the ACT clinic spayed or neutered 1,701 cats during normal hours in that year. It continued to offer a once-a-month Spay Day, helping another 1,018 cats in 2006. As mentioned above, one of the challenges of free-roaming cat surgeries is the availability of trained staff to provide care for the cats when they come in during regular hours. ACT took free-roaming cats with no reservation necessary every day the clinic was open. The methods and medical protocols followed by these clinics are documented by [Looney et al. (29)] and by Griffin et al. (30).

**Figure 2:**
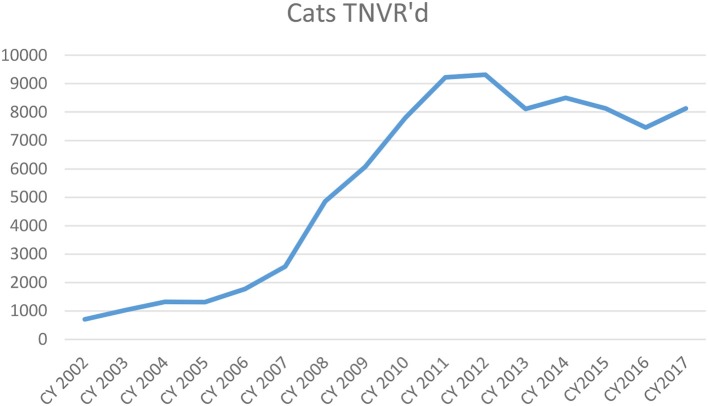
Free-roaming cats TNVR Surgeries by ACT and HSTB.

As the ACT program grew, HSTB also tried to increase its spay/neuter efforts for free-roaming cats. As Figure 2 shows, the number of community cats sterilized increased between 2002 and 2012 as both clinics focused on large-scale efforts. Using Mission Orange funds, HSTB hired a full-time TNVR coordinator. She controlled the loaned traps and organized trappers to help citizens trap free-roaming cats.

There was some dissention among the various parties involved in efforts to expand TNVR to control what the AAC called “community cats.” Some local veterinarians, including the HCVMA and HAHF, along with some dog rescue groups wanted the effort stopped. This group's stance was that TNVR was illegal and posed a human health danger. A Citizen's Initiative on Community Cats was proposed to the BOCC in June 2011 by concerned trappers and caretakers. And the ASPCA for its part supported continuing efforts to conduct large-scale TNVR. The upshot was that on December 7, 2011, the BOCC passed a resolution recognizing that there was a community cat population continually producing offspring and noting that TN[V]R had been recognized by national organizations as a way of trying to manage the problem. The resolution further said that “the BOCC also recognizes TN[V]R programs… that both comply with federal, state, and local laws [and] with the guidelines of the ASPCA and HSUS for TN[V]R, as another means to reduce the community cat population in addition to trapping and euthanizing” (31).

In 2012 HSTB opened a larger animal hospital and reserved every Monday for treating free-roaming cats at low cost. It designed a cat patio that allowed trappers to drop off their cats in traps on Sundays and pick them up after surgery on Monday afternoons.

The BOCC took a further step toward supporting TNVR when in May 2013 it endorsed the “Be the Way Home” plan (32) developed by a county task force and the new Animal Services director, outlining 60 separate initiatives to increase the number of animals leaving the Animal Services shelter alive. The initiatives were divided into eight categories and covered all facets of shelter operations (marketing, volunteering, technology, revenue, intake, spay/neuter, adoptions and rescues, and return to owner). An ordinance (No. 13-33) passed in December 2013 provides the legal framework within which community cat programs still operate in the county.

A number of issues that caused controversy among interested groups and agencies as the “Be the Way Home” programs were implemented are addressed below in section The Opposition, discussing opposition to free-roaming cat management programs in the county.

### Return-to-Field (RTF)

In 2008 the city of Jacksonville, in northeast Florida, started Project Feral Freedom, which targeted community cats admitted to a shelter. The project was the product of a close working relationship between a community group, First Coast No More Homeless Pets (FCNMHP), and the city. FCNMHP initially asked the director of Animal Care and Control for the city if it could pick up any ear-tipped cats turned into the Jacksonville shelter and return them to where they were picked up. The director's response was that FCNMHP could take all the free-roaming cats at the shelter (33), which it began to do in August 2008. FCNMHP picked up the cats, treated them, and returned them to where they were picked up. This was the genesis of Return to Field (RTF).

Other cities, including San Jose, Charleston, SC, San Antonio, Albuquerque, Baltimore, Philadelphia, Tucson, and Columbus, GA subsequently started similar programs (33–36). Hillsborough County decided to undertake its own Project Feral Freedom program in 2014.

RTF is the most radical of the three programs discussed in this paper because there was no known caretaker for the cats to go back to after sterilization. This arrangement was rationalized on the basis that the returned cats already had a home. It was not what people usually understood as a home, but the cats involved were thriving and healthy for the most part, which meant that they had found food sources and shelter close to where they were picked up. In that sense they did have a home (37).

As noted in Table 1, free-roaming cats (defined as strays by HCAS/PRC) make up a large percentage of the total intake and workload of the Hillsborough County shelter. Although the percentage of free-roaming cats taken in remained about the same from 2006 to 2017 (between 35 and 54%), total cat intake dropped by more than half over the same period.

It is hard to gauge the precise impact of the RTF program in Hillsborough County. While it is a targeted program aimed at a specific subset of healthy, adult, non-owned, free-roaming cats that are admitted to the only open-access shelter in the county, it is a small-scale effort (as shown in Table 2), chiefly because of funding constraints. In fact, there is at the moment no county funding for RTF. The cats are identified upon entry to HCAS/PRC and transported to HSTB for sterilization and shots, paid for by HSTB. Volunteers transport the cats from HSTB back to where they were captured and release them. Data on the cats was kept both by HCAS/PRC and HSTB. By way of comparison, between 2010 and 2014, in San Jose, California, a community of over a million people, 10,080 free-roaming cats were admitted to the animal shelter and treated prior to release, all at municipal expense. It is conceivable that if the Hillsborough County program were supported in the same way, it could achieve a higher than 90% live-release rate (LRR). The actual rate for cats in 2018 was 85.5%. Live release rate (LRR) is defined as live outcomes divided by intake [(38), p. 6], expressed as a percentage. In 2005 the LRR for HCAS (the only open-access shelter in the county) was 4.6%, indicating an increase of 80.9% by 2018.

**Table 2:** Cats returned to field in Hillsborough County.

**Calendar year**	**Returned to field**
2014	1,015
2015	730
2016	829
2017	1,344
Total	3,918

Return-to-field programs are different from TNVR programs because they involve free-roaming cats that have been admitted to a shelter. This makes them part of the animal shelter and control system (39). They have been trapped by either animal control officers or members of the public. In the past, when cats were admitted to a shelter, they faced the almost certain prospect of being euthanized. Against this, the RTF alternative provides the hope that for suitable, healthy, free-roaming cats there will be a better outcome. The Million Cat Challenge–an initiative launched in 2014 by the shelter medicine programs at the University of California, Davis and the University of Florida veterinary schools to save the lives of one million cats over 5 years–offered this perspective on RTF: “No greater harm to communities is caused by returning shelter cats to their neighborhoods with the benefit of birth control and vaccines, and much is gained by engaging the community in a positive response” (37).

## Other Factors Affecting Free-Roaming Cat Management

### Social Media

During the time that free-roaming cat management programs have been evolving in Hillsborough County there has been a dramatic and universal change in communications technology, a change that has mediated one of the most persistent problems that stands in the way of making free-roaming cat management a success, not just in this one county but more generally: how to find and connect people who will support such programs across the country. It is worth recalling that the first iPhone was released in 2007. From January 2007 to December 2014, according to AT&T, mobile data traffic increased by more than 100,000% [(40), p. 20]. Change.org, the most popular social mobilization website, also came to life in 2007. The ability to share information and images easily across platforms and networks, particularly through user-generated content, has had a major and positive impact on the animal welfare community. It facilitates the organizing of like-minded individuals. It allows people to contact each other easily and quickly about free-roaming cats in need of help, including their pictures, their locations, their numbers and, if they are in a shelter, their likely time to euthanasia. And it makes it easier to raise money, both for medical expenses and for general support (41).

National online communities such as Maddie's Pet Forum, Out the Front Door, and Vox Felina provide relevant information and let people ask questions. This gives local activists and caretakers a largely unconstrained avenue for both learning and connection. The Million Cat Challenge and Out the Front Door websites, ASPCA position statements, and open-source articles such as Spehar and Wolf's 2018 and 2019 papers on RTF and TNR are now readily available to all the stakeholders in the national conversation about free-roaming cat management, which means that no single group of stakeholders can now control that conversation.

So whereas in 1997 caretakers for free-roaming cats were essentially an underground resource and tried to remain hidden so that the cats would not be taken by their neighbors or animal control, they are now visible and organized and connected through listserves and other social media devices.

In Hillsborough County specifically, caretakers for free-roaming cats were reached and connected through social media by HSTB and ACT. The Tampa Bay Cat Rescuers' Facebook page, for example, has attracted 4,176 readers and followers (42).

This huge change in the ability of people who care about free-roaming cat management issues to be connected and to be engaged and to share information made its influence felt when the “Be the Way Home” plan was presented to the BOCC. More than 200 people showed up for the deliberations, many wearing identical green t-shirts to demonstrate their support for the plan to the county commissioners.

### The Opposition

Opposition to free-roaming cat programs has come in the past from local and state governments, from some veterinarians, even from some animal welfare advocates, and from citizens who are worried about the impacts free-roaming cats might have on wildlife, especially birds. One opposition strategy has been for wildlife officials to assert that free-roaming cats are a form of wildlife and can therefore be regulated by wildlife protection agencies as a threat to other and more valuable species. Although the FWC finally stated that free-roaming cats were not considered wildlife, these claims that TNVR is illegal continue (43, 44).

Opposition to any change to the status quo in the county started with the discussion of the SNVP from 2002 to 2006. The principal opposition to free-roaming cat management in Hillsborough County surfaced in 2012 and 2013 when a task force was at work to consider options and when the “Be the Way Home” plan was being developed. The opposition was rooted in earlier efforts against the launch of the Citizens Initiative Community Cat Management Program in March 2011, when the HCVMA and HAHF helped organize other animal advocates (“dog people”) into a citizens group. This group did not really understand cat issues and were heavily influenced by the fact that their veterinarians favored euthanasia of all free-roaming cats. Group members and those veterinarians spoke out against any plans aimed at nonlethal management of free-roaming cats during the many meetings during this time period.

During the debate on the task force report, a plan called AWAKE (43) was proposed suggesting that the county provide land, with no electricity or running water, to house a sanctuary for free-roaming cats to avoid creating a public health hazard. The assumption was that volunteers caring for free-roaming cats would be willing to drive to the southern part of the county to take care of them. The plan also anticipated weekly visits from a paid veterinarian and vet tech, but with no mention of how the plan would be financed. This plan is still accessible on the HAHF website. National groups such as Best Friends and Alley Cat Allies came out against the plan (45). Although the opposition has abated over the past few years, if any free-roaming cat is found to have rabies, the opponents of TNVR are revitalized (46, 47).

## Results and Discussion

The three targeted programs were integrated into the daily operations of the two clinics. Spay/neuter surgeries due to the first two programs started ramping up in 2002 along with the volume of spay/neuter surgeries for all cats and peaked in 2011. The RTF surgeries started in 2014. In the aggregate, over the period analyzed (2002–2017) more than 38,000 SNVP cat surgeries were performed, 86,000 TNVR surgeries were performed by ACT and HSTB (Figure 2), and 3,918 RTF surgeries were performed (Table 2). Volume and consistency are critical to the success in assisting in lowering the numbers of cats flowing into the shelter and subsequently being euthanized.

**Figure 3:**
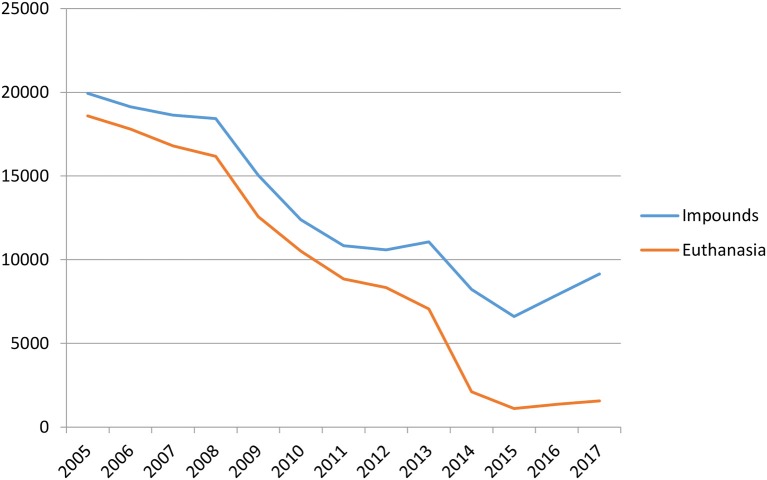
Hillsborough County Animal Services cat intake and euthanasia, 2005–2017.

Figure 3 shows that until 2011–2012, the first two targeted programs (SNVP and large scale TNVR) jointly developed by the county and cat welfare groups for reducing the number of cats entering the shelter was working. Cat intake numbers were dropping; however, the chances of live release remained low. The LRR increased by only 8% from 2001 to 2010. The LRR in 2001 was 5.7%. It dropped to 4.6% by 2005 and then climbed slowly to 13.4% in 2010 (HCAS/PRC annual reports). Marsh (17) noted that there was a correlation (*r* = 0.986) between shelter intake and euthanasia in Hillsborough County from 1997 to 2009 (p. 7). After 2012, with a push from a new animal services director and mobilized citizens, the rate steadily increased as reduced intake and a focus on live releases became county policy. The three targeted programs discussed above helped to bring about this shift. The rise in intakes in 2017 might be explained by the decrease in the number of low-cost vouchers issued and redeemed between 2015 and 2017, or by the fact that shelter hours for drop-off and intake by members of the public increased from 20 h a week in 2014 to 54 h a week in 2016. Because companion animal population management is a dynamic and complex problem, it is hard to be sure which variables explain most of the variance in the data. Other factors, including changes in shelter procedures, can also influence the numbers of cats flowing into the shelter and subsequently being euthanized. During this time period, four shelter directors were at the helm. Each changed procedures that could affect intake such as intake diversion, changing officers' duties, and shelter hours. Each of these has to be done with the consideration that abandonment may increase if you make it too hard for the citizens.

It is reasonably clear, however, that the citizens of Hillsborough County had three choices in the early 2000s. They could continue to live with an ongoing free-roaming cat problem. They could wait for the government to solve the problem. Or they could try to organize and mobilize a diverse set of skills in the local community to change the situation. Over the period of this study, they chose to change to nonlethal means of companion animal population control.

This empirical study of Hillsborough Country, Florida, demonstrates that there are several things a community can do to increase the live-release rate of cats from open access shelters. The first is to attempt to reduce shelter intake by performing affordable and accessible spay and neuter surgeries on two target populations: cats owned by low-income families and free-roaming cats. The second is to identify innovative techniques to return greater numbers of sterilized free-roaming cats to the field. The third is to get the entire community involved in any effort to improve the LRR from the open access shelter. Johnson and Cicirelli (34) report that impounds of cats and kittens in San Jose decreased from 70% of all intakes in 2010 to 23% in 2014 and that shelter euthanasia for cats suffering from feline upper respiratory infections decreased by 99%. Although comparable numbers are not available for Hillsborough County, feline intake decreased by 51% over a period of about a decade (see Table 1), even while the county population and the number of their pets increased substantially. The number of households in the county increased by 119,623 from 2004 to 2017, an increase of 26.9% (14, 48). According to the American Veterinary Medical Association, 30.4% of households own 2.1 cats (49). This is presumptive evidence that the three programs discussed in this analysis have had a positive impact. In 2017, the Hillsborough shelter took in 9,151 cats and had a live-release rate of 81.8 % (7,589). That is a notable achievement.

Advocates of better management for free-roaming cats need to be aware that it takes organization, leadership, and determination to adopt and implement new programs in the face of opposition. Those who can document their successes need to share what they have learned, and one of the goals of this paper is to contribute to such sharing of information.

Finally, the technological revolution that has provided new means to share information has connected the world more than ever before (40). A large number of citizens do not want their community to kill animals as a means of population control. Using technology to share innovative ideas and successful methods will ensure that these programs will be replicated.

## Conclusions

Robertson wrote in 2008, “Feral cats are a result of human actions; we caused the problem and we should be responsible for a solution” (p. 373). Five years later, the Alliance for Contraception in Cats and Dogs (ACC&D) was able to say, “If TN[V]R is performed with sufficient intensity and for a sufficient duration, it can be effective in reducing population size, as long as dispersal (newly abandoned cats or other cats immigrating) into the treated population does not exceed a defined threshold level” (50). The three targeted programs introduced in Hillsborough County have operated at a fairly high level of intensity. Almost 128,000 targeted cat surgeries were performed along with other untargeted surgeries.

In the future, the likelihood is that new methods of high-volume, free-roaming cat reduction will be developed and that they will rely less than they do now on the work of volunteers. There are already 18 Humane Alliance clinics in Florida with a capacity for high-volume work and other local Humane Societies and SPCAs are adopting these methods.

## Author Contributions

The listed author participated as a member of the Animal Advisory Committee and co-founder of one of the participating organizations. FH gathered the material, interviewed participants, and wrote the paper.

### Conflict of Interest Statement

The author cofounded Animal Coalition in 2001 and sat on the Animal Advisory Committee (AAC) from 2001 to 2009. FH is not currently employed in any capacity in the animal welfare community.

## Footnotes

^1^Hillsborough County Animal Advisory Committee (n.d.). Available online at: https://www.hillsboroughcounty.org/en/government/boards-and-committees/a-d/animal-advisory-committee

^2^Hillsborough County Animal Services Internal Financial Working Papers (n.d.). Animal Advisory Committee Handout.
